# Preoperative chemotherapy prior to pulmonary metastasectomy in surgically resected primary colorectal carcinoma

**DOI:** 10.18632/oncotarget.2172

**Published:** 2014-07-08

**Authors:** Ishwaria M. Subbiah, Shanda H. Blackmon, Arlene M. Correa, Bryan Kee, Ara A. Vaporciyan, Stephen G. Swisher, Cathy Eng

**Affiliations:** ^1^ Division of Cancer Medicine, The University of Texas MD Anderson Cancer Center, Houston, Texas; ^2^ Department of Thoracic and Cardiovascular Surgery, The University of Texas MD Anderson Cancer Center, Houston, Texas; ^3^ Department of Gastrointestinal Medical Oncology, The University of Texas MD Anderson Cancer Center, Houston, Texas; ^4^ Department of Surgery, The Methodist Hospital and Weill Cornell College of Medicine, Houston, Texas

**Keywords:** colorectal, carcinoma, neoadjuvant, chemotherapy, metastasectomy

## Abstract

**Background:**

The benefit of preoperative chemotherapy prior to pulmonary metastasectomy for patients with colorectal carcinoma (CRC) is unknown. Here, we identify outcomes of preoperative chemotherapy in patients with resected primary CRC who then underwent pulmonary metastasectomy.

**Methods:**

We queried a prospective database to identify treatment characteristics. Multivariate analyses identified predictors of overall survival (OS) and progression-free survival (PFS).

**Results:**

229 patients underwent lung metastasectomy, of whom 115 proceeded to surgery without chemotherapy while 114 received preoperative regimen based on oxaliplatin (32%), irinotecan (46%), capecitabine (16%), or other (6%). Median PFS in preoperative chemotherapy vs. surgery alone arms were comparable (p=0.004). Patients on oxaliplatin-based therapy had an improved OS vs. an irinotecan, capecitabine, or alternate regimen (p=.019). On multivariate analysis, the irinotecan subset had a worse OS (HR 1.846; 95% CI 1.070, 3.185) vs. surgery alone arm (p=0.028). The OS of an oxaliplatin-based regimen vs. no chemotherapy was inconclusive (HR 0.57; 95% CI 0.237 to 1.389, p=0.218). Multivariate analysis demonstrated a worse PFS and OS for the male gender and an incomplete resection (R2).

**Conclusion:**

Prospective trials on specific preoperative regimens and criteria for patient selection may identify a role for preoperative chemotherapy prior to a curative pulmonary metastasectomy.

## INTRODUCTION

Pulmonary recurrence of colorectal cancer (CRC) following resection of the primary CRC lesion presents a significant clinical problem because the lung is a common site of extracolonic recurrence [1, 2]. The optimal sequence of management of pulmonary oligometastatic disease remains unclear, although emerging data indicate a role for surgical resection in patients with metastases in the lung alone. Surgical intervention for this subset of patients has been explored with reports of 11% to 41% probability of 5-year survival in patients with resection of oligometastatic pulmonary lesions [3-5]. Indeed, the significant improvement in survival after resection of pulmonary metastases in the absence of extrapulmonary disease lesions has led to the widespread clinical practice of surgical resection in these patient populations [1, 3, 5, 6]. Furthermore, improved progression-free survival (PFS) and overall survival (OS) has been noted in patients who have undergone lung metastasectomy compared to those who deferred surgery [7]. Similarly data even suggests that lung metastases are not a poor prognostic factor for survival in patients undergoing resections for both liver and lung metastases, compared to liver metastases alone [8]. However, most analyses are retrospective single institution experiences and report a wide range of 5-year survival ranging from 21% to 61%. Furthermore, OS was associated with an elevated CEA level, tumor location (unilateral vs. bilateral), and number of pulmonary metastases [9-13]. Preoperative predictors of prognosis may identify subsets of patients who may benefit from a multimodal therapeutic strategy incorporating preoperative chemotherapy prior to a potentially curative resection of oligometastatic disease. The actual benefit of preoperative chemotherapy prior to pulmonary metastasectomy, as well as the optimal regimen, are not well defined and require further analysis. Consequently, we investigate the characteristics, treatment regimens, and outcomes of patients with resected primary tumors who then underwent pulmonary metastasectomy.

## RESULTS

### Patient characteristics

We identified 8,712 patients with CRC who were evaluated at MD Anderson from January 1, 2000, to December 3, 2010; of these patients, 2,595 had metastases to the lung. We characterized 229 consecutive patients who underwent pulmonary metastasectomy at our institution. Patient characteristics and description of disease status are listed in Table 1. Of these 229 patients, 115 (50%) underwent surgical resection alone while 114 (50%) received preoperative chemotherapy prior to resection of the pulmonary lesions. Of the 115 patients in the surgery alone arm, 85 patients had early stage disease (defined as any T, any N, M0) of colorectal cancer at the time of their initial diagnosis while 24 patients had stage IV disease (any T, any N, M1); in the preoperative chemotherapy arm, 68 patients had early stage colorectal cancer at the time of diagnosis while 40 patients had stage IV disease. The initial disease status was unknown for 6 patients in each arm. Seventeen of 38 (45%) patients in the preoperative chemotherapy arm who underwent molecular testing demonstrated a *KRAS* mutation in codons 12, 13, and 61. Similarly 14 of 32 (44%) tested patients in the surgery alone arm had an identified *KRAS* mutation. The median time to diagnosis of metastatic disease from time of resection of the primary colorectal tumor to the time of resection of the pulmonary metastases was 35.1 months (range, 1.0 – 143.4 months) in the preoperative chemotherapy arm and 34.1 months (range, 2.2 – 149.2 months) in the surgery alone arm. Of the 115 patients who proceed with surgery without preoperative chemotherapy, the median number of metastatic lesions within the lung was 1 (range, 1-7) and the median size of the largest lung nodule was 1.4 cm (range, 0.3 – 10 cm). In the preoperative chemotherapy arm, the median number of pulmonary lesions was 2 (range, 1 – 16) with the median size of the largest nodule being 1.8 cm (range, 0.2 – 14.5 cm). Baseline characteristics did vary significantly among patients receiving the four groups of preoperative regimens (Table 2). Specifically, a greater number of patients who received preoperative chemotherapy prior to pulmonary metastasectomy had stage IV disease at time of initial colorectal cancer diagnosis (p=0.015), more than two pulmonary metastases (p<0.001), and a greater size of largest lung metastatic lesion (p=0.009).

**Table 1:** Baseline patient and operative characteristics of surgery alone and the preoperative chemotherapy arms

	Surgery Alone Arm	Preoperative Chemotherapy Arm	p value
N	115	114	
Median age at time of pulmonary metastasectomy, years	62 (95% CI 50.3, 73.7)	59 (95% CI 48.3, 69.7)	0.178
Gender			0.203
Male	60 (52%)	69 (61%)	
Female	55 (48%)	45 (39%)	
Stage of colorectal cancer at time of diagnosis			0.015
Early Stage (anyTanyNM0)	85 (74%)	68 (60%)	
Stage IV (anyTanyNM1)	24 (21%)	40 (35%)	
Unknown	6 (5%)	6 (5%)	
Number of lung metastases			
≤ 2 lesions	102 (89%)	66 (58%)	<0.001
> 2 lesions	13 (11%)	48 (42%)	
Size of largest lung mass (cm), median	1.3 (95% CI 0.1, 2.5)	1.8 (95% CI 0.01, 3.6)	0.009

**Table 2:** Disease characteristics across the four preoperative chemotherapy regimens among 114 patients receiving preoperative chemotherapy

	Oxaliplatin	Irinotecan	Capecitabine	Other
N	37	52	18	7
Age (years) at time of resection, median	58	59	62	62
Duration (months) of preoperative chemotherapy, median	3.5 (95% CI 0.5, 6.5)	4.4 (95% CI 0.7, 8.1)	4.1 (95% CI 2.0, 6.2)	3.3 (95% CI 1.2, 5.4)
Median # of lung metastases	2	3	2	3
Size of largest lung metastases, median (cm)	1.6 (range 0.5, 5.5)	1.7 (range 0.2, 9.3)	1.9 (range 0.8, 4.5)	2.3 (range 1.3, 4.8)
Presence of synchronous liver metastases	15 (41%)	31 (60%)	9 (50%)	6 (86%)
Type of resection at pulmonary metastasectomy				
R0	24 (65%)	35 (67%)	12 (67%)	3 (43%)
R1	8 (21.6%)	6 (12%)	4 (22%)	2 (28.5%)
R2	5 (13.5%)	11 (21%)	2 (11%)	2 (28.5%)
Differentiation of tumor on pathology				
Well differentiated	1 (3%)	0 (0%)	0 (0%)	0 (0%)
Moderately differentiated	29 (78%)	42 (81%)	17 (94%)	6 (86%)
Poorly differentiated	7 (19%)	10 (19%)	1 (6%)	1 (14%)

### Preoperative chemotherapy regimen

The median duration of preoperative therapy was 4.1 months (95% CI 2.4, 6.5). A total of 114 patients received preoperative chemotherapy: 41 patients (36%) received 0 to 3 months of preoperative chemotherapy; 37 patients (32%) received 3 to 6 months; 36 patients (32%) received greater than 6 months. Biologic therapy was provided to a total of 67 (59%) patients in combination with a cytotoxic agent; specifically, 37 (32%) patients received an oxaliplatin-based regimen (including FOLFOX) of which 24 patients (21%) received this regimen in combination with bevacizumab; 3 patients received FOLFOX with cetuximab and 1 patient received FOLFOX with an investigational agent. Fifty-two (46%) patients received an irinotecan-based regimen; 23 patients (20%) in combination with bevacizumab, 4 with cetuximab, and 3 with other biologic agents. Eighteen (16%) patients received a capecitabine regimen, of which 6 was in combination with bevacizumab. Finally, 7 patients received other systemic regimens preoperatively including investigational targeted therapies. The surgical outcomes for all patients groups are outlined in Tables 2 and 3

**Table 3:** Surgical outcomes of all patients undergoing a pulmonary metastasectomy

	Surgery alone arm	Preoperative chemotherapy arm	p value
N	115	114	
Initial pulmonary metastasectomy			0.109
R0 resection (%)	89 (77%)	74 (65%)	
R1 resection (%)	14 (12%)	20 (18%)	
R2 resection (%)	12 (11%)	20 (18%)	
# of patients with recurrence in the lung after initial pulmonary metastasectomy	37 (32%)	34 (30%)	0.832
# of 2^nd^ pulmonary metastasectomy	28 (76%)	24 (71%)	
# of 3^rd^ pulmonary metastasectomy	9 (24%)	10 (29%)	

### Postoperative therapies after pulmonary metastasectomy

Of the 115 patients in the surgery alone arm, 44 patients (38%) received chemotherapy postoperative after pulmonary metastasectomy. The median time from surgery to initiation of chemotherapy postoperatively was 1.5 months (95% CI 1.2, 1.8). Overall, 38 (33%) patients were treated in the absence of disease aiming to reduce the risk for disease recurrence. The remaining six patients showed evidence of active disease on their first postoperative imaging with four patients developing new pulmonary lesions, one patient with a new liver lesion, and one with new intra-abdominal adenopathy and rising CEA. Postoperatively, 19 patients received an oxaliplatin-based therapy, 13 an irinotecan containing regimen, and 11 a capecitabine-regimen. Sixteen patients received bevacizumab in conjunction with a systemic chemotherapy regimen, and two received cetuximab, one of which was as a single agent.

Of the 114 patients who received preoperative chemotherapy prior to pulmonary metastasectomy, 54 (49%) patients received chemotherapy or radiation within 3 months postoperatively; among these 54 patients, the median time from surgery to resumption of chemotherapy postoperatively was 1.5 months (95% CI 1.4, 1.7). In 37 (32%) patients, postoperative therapy was given as adjuvant therapy in the setting of no active disease with the intention of extending the recurrence-free period. The remaining 17 patients demonstrated evidence of active disease on postoperative evaluation which prompted the initiation of additional chemotherapy or radiation. Specifically, 4 patients had positive surgical margins, 5 patients had new lesions in the lungs on their first postoperative imaging. Three patients developed new hepatic lesions including one patient with concurrent new hepatic and pulmonary masses. Two patients developed enlarging lymph nodes. One patient each developed either a local recurrence at the anastomotic site of the initial colonic resection, new bone metastases, or a rising CEA.

Of the 54 patients receiving postoperative therapy, two patients received consolidative radiation therapy: one to the positive surgical margin and a second to the regional thoracic lymph node basin where one excised node was positive for malignancy. A third patient underwent radiofrequency ablation to a solitary liver lesion. The remaining 51 patients received systemic therapy, most commonly with a regimen based on irinotecan- (n=22), oxaliplatin (n=18), or capecitabine (n=9). Bevacizumab (n=19) or an anti-epidermal growth factor receptor (EGFR) targeting therapy (cetuximab or panitumumab, n=11) was given concurrently with a cytotoxic regimen.

### Survival Analysis

The median time of follow-up after the initial pulmonary metastasectomy was 33.1 months (95% CI 24.6, 41.6) in the preoperative chemotherapy arm and 42.3 months (95% CI 34.6, 50.0) in the surgery alone arm. The median PFS among the 115 patients in the surgery alone arm was 18.1 months (95% CI 14.6, 21.6) while the preoperative chemotherapy arm demonstrated a median PFS of 18.8 months (95% CI 15.1, 22.6). Of the 4 different chemotherapy arms, the median PFS was 18.2 months (95% CI 14.2, 22.2) in the irinotecan-based therapy arm, 19.6 months (95% CI 11.3, 27.9) in the oxaliplatin arm, 20.8 months (95% CI 12.0, 29.5) in the capecitabine arm, and 27.7 months (95% CI 1.0, 54.4) among patients receiving other systemic therapies.

**Figure 1:**
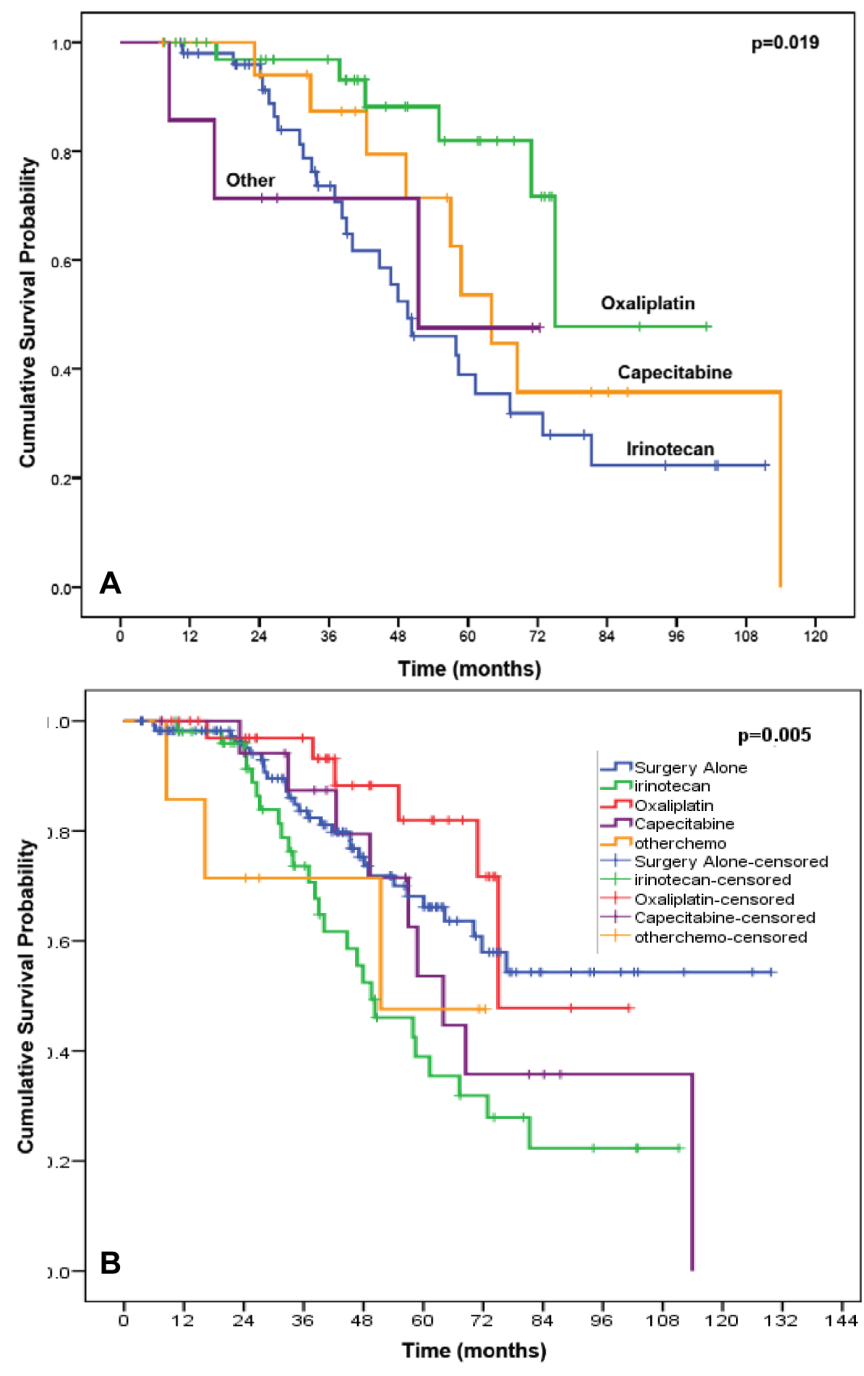
Kaplan-Meier curve demonstrating that the overall survival of patients in the 4 preoperative chemotherapy arms (p<.019), using the irinotecan-based therapy group as reference (A) and the surgery alone group as the reference (B).

For overall survival the median OS was not reached among patients in the surgery alone arm subset (mean OS 91.7 months, 95% CI 80.9, 102.5). Among the 4 preoperative chemotherapy arms, patients receiving an irinotecan-based regimen represented the largest subset among our patients receiving preoperative chemotherapy, thereby serving as the reference group. This irinotecan-receiving subset had a worse OS (49.6 months; 95% CI 35.3, 63.9) in comparison to any chemotherapy regimen (p 0.024). Patients receiving an oxaliplatin-based therapy demonstrated an improved OS in comparison to an irinotecan-based therapy group with a mean OS 81.0 months (95% CI 67.7, 94.4, p = 0.019) (Figure 1); the median OS has not yet been reached in the oxaliplatin-group. Patients receiving a capecitabine-based regimen demonstrated a median OS of 64.0 months (95% CI 52.9, 75.1) while patients on other systemic therapies did not reach median OS (mean OS 50.3 months, 95% CI 30.7, 69.8).

**Figure 2:**
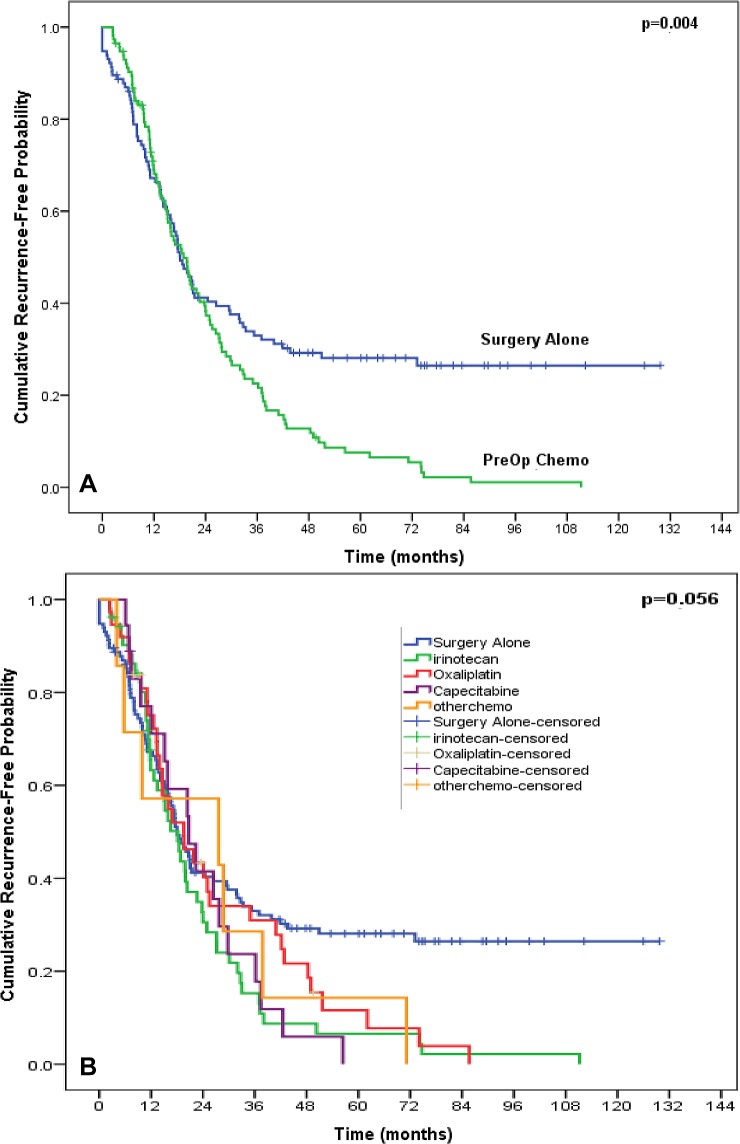
Kaplan-Meier curve of progression-free survival comparing the surgery alone arm versus overall preoperative chemotherapy arm (A) and each of the 4 subsets of preoperative chemotherapies (B).

The 67 patients who received a biologic drug (bevacizumab, cetuximab, or panitumumab) as part of their treatment regimen had a median PFS of 13.5 months (95% CI 10.8, 16.2) in comparison to the 47 patients who did not receive a biologic agent (median PFS 24.9 months; 95% CI 18.4, 31.4). However the median OS did not differ significantly between these two groups (p=0.144). Receiving a bevacizumab-based regimen did not significantly impact median OS (51.5 months; 95% CI 42.8, 60.2) when compared to patients receiving a preoperative regimen that did not include bevacizumab (55.1 months; 95% CI 48.9, 61.4, p=0.435).

On multivariate Cox regression analysis accounting for age at time of pulmonary metastasectomy, gender, size of largest pulmonary metastases, number of pulmonary metastases and type of resection (R0, R1, or R2), the PFS did not vary significantly among the 4 arms of preoperative chemotherapy when compared to the surgery alone arm (p=0.264) (Table 4). However, on multivariate analysis of OS, the irinotecan-based chemotherapy group did demonstrate a worse OS (hazard ratio [HR] 1.846; 95% CI 1.070, 3.185; p=0.028) in reference to the surgery alone arm (Table 5). The oxaliplatin-based chemotherapy group trends in favor of an improved OS (HR 0.574; 95% CI 0.237, 1.389) when compared to the surgery alone arm (without chemotherapy) but did not reach statistical significance (p=0.218).

**Table 4:** Multivariate cox regression analysis of progression free survival across the surgery alone arm and the 4 arms of preoperative chemotherapy regimen

			95% CI for HR		
	Frequency	Hazard Ratio	Lower	Upper	p-value
Type of intervention for lung metastases					0.264
Surgery Alone (Reference)	115	1.000			
Irinotecan-based chemotherapy	52	1.379	0.937	2.029	0.103
Oxaliplatin-based chemotherapy	37	1.235	0.812	1.876	0.324
Capecitabine-based chemotherapy	18	1.459	0.853	2.493	0.168
Other systemic therapy	7	0.748	0.273	2.047	0.572
Gender					
Female (Reference)	100	1.000			0.036
Male	129	1.379	1.021	1.862	
Type of pulmonary resection					0.045
R0 (Reference)	163	1.000			
R1	34	1.139	0.734	1.767	0.561
R2	32	1.780	1.131	2.803	0.013

HR hazard ratio. Adjusted for age at time of pulmonary metastasectomy, gender, size of largest pulmonary lesion, # of lung metastases and type of resection.

**Table 5:** Multivariate cox regression analysis of overall survival across the surgery alone arm and the 4 arms of preoperative chemotherapy regimen

			95% C.I. for HR		
	Frequency	Hazard Ratio	Lower	Upper	p-value
Type of intervention for lung metastases					0.054
Surgery Alone (Reference)	115	1.000			
Irinotecan-based chemotherapy	52	1.846	1.070	3.185	0.028
Oxaliplatin-based chemotherapy	37	0.574	0.237	1.389	0.218
Capecitabine-based chemotherapy	18	1.600	0.756	3.389	0.219
Other systemic therapy	7	1.525	0.435	5.347	0.509
Gender					
Female (Reference)	100	1.000			0.016
Male	129	1.921	1.130	3.267	
Type of pulmonary resection					0.012
R0 (Reference)	163	1.000			
R1	34	1.062	0.523	2.155	0.868
R2	32	2.802	1.412	5.561	0.003

Adjusted for age at time of pulmonary metastasectomy, gender, size of largest pulmonary lesion, # of lung metastases and type of resection.

The multivariate analysis also revealed a gender predilection favoring women with men undergoing pulmonary metastasectomy demonstrating a worse PFS (HR 1.379; 95% CI 1.021, 1.862; p=0.036) and OS (HR 1.921; 95% CI 1.130, 3.267; p=0.016). Similarly, patients who underwent an R2 resection demonstrate a poorer PFS (HR 1.780; 95% CI 1.131, 2.803; p=0.013) and OS (HR 2.802; 95% CI 1.412, 5.561; p=0.003) when compared to an R0 resection; no difference of significance was noted in PFS (HR 1.139, p=0.561) or OS (HR 1.062, p=0.863) among patients who underwent an R1 resection when compared to an R0 resection.

## DISCUSSION

Aggressive surgical intervention in pulmonary oligometastatic disease has shown in previous retrospective non-randomized studies to provide a 5-year survival as high as 50% with a subset of patients being cured after resection of their pulmonary metastases [2, 3, 6, 13-15]. However, the exact role for preoperative chemotherapy with surgically resectable pulmonary oligometastatic disease in CRC patients has not been previously established. The characteristics of treatment failure after pulmonary metastasectomy where there is subsequent recurrence of disease may suggest that a multimodality approach may potentially improve the duration of recurrence-free survival in these patients [10, 16-18]. Our exploratory analysis suggests a potential role for an oxaliplatin-based regimen if preoperative therapy is considered prior to a potentially curative pulmonary metastasectomy thereby improving overall survival benefit in comparison to an irinotecan or capecitabine-based regimen. In the current study, the use of bevacizumab did not provide any additional benefit.

Patient selection for preoperative chemotherapy prior to resection of lung metastases remains an area of investigation. One independent analysis of our current study's patient population investigated clinical predictors of recurrence in the lung among patients after their initial CRC pulmonary metastasectomy; this work has demonstrated increasing number of lung metastases and a shorter preoperative disease free interval as predictors of increased risk for lung recurrence [12]. Furthermore, the same analysis characterized increasing age (greater than 60 years) and the male gender to independently predict shorter survival with the lowest overall survival identified among men older than 60 years of age with greater than 3 metastatic lesions in the lung (35.7 months, P <0.001) [12]. Indeed, the number of pulmonary metastases has predicted for shorter overall survival in several large series [9, 11, 18, 19]. Indeed our analysis is limited by unmeasured confounding variables including performance status and comorbidities and their potential role in influencing patient selection for preoperative chemotherapy prior to a curative pulmonary metastasectomy. Additional factors limiting our analysis include the single institution, retrospective nature of the study in which characteristics of the disease and chemotherapy regimen was captured. However, there is homogeneity in the surgical selection for this patient population.

## CONCLUSION

This exploratory analysis provides insight regarding possible therapeutic approaches to this subset of patients with oligometastatic disease in the lung with features portending for a shorter PFS and OS. Additionally, there is little literature that has clearly defined the appropriate multimodality approach to this patient population. The findings of favored overall survival after having received an oxaliplatin-based regimen when compared to no preoperative chemotherapy, remains to be investigate. Upcoming trials on specific preoperative regimens and criteria for patient selection are needed and planned. An ongoing prospective phase III trial is the first such to randomize patients with a prior resected colorectal cancer to active monitoring or active monitoring with pulmonary metastasectomy; primary endpoints include overall survival, relapse-free survival, and patient-reported quality of life [20-24]. Another planned prospective phase II study (ACCLAIM) also aims to address this very issue with concurrent molecular correlatives. Further investigation into patient characteristics may reveal patterns of recurrence that can facilitate patient selection for the most appropriate preoperative chemotherapy. Furthermore, molecular marker analysis may provide additional information regarding the primary tumor, site of metastatic disease or disease recurrence. We propose prospective pulmonary metastasectomy trials incorporating such selection may identify the subset of patients most likely to benefit from multidisciplinary approach to pulmonary oligometastatic disease.

## MATERIALS AND METHODS

We queried a prospectively maintained thoracic surgery database at the University of Texas MD Anderson Cancer Center for patients with CRC who underwent their first pulmonary metastasectomy at our institution from January 1, 2000, to December 3, 2010. Eligibility criteria included the presence of unilateral or bilateral resectable lung lesions on preoperative CT scan of the chest without local colorectal recurrence or distant extrahepatic disease; the primary colorectal tumor was surgically removed with an R0 resection; treatment-naïve to systemic chemotherapy prior to lung resection. Prior resected or patients who had surgically resectable hepatic metastases were not excluded. Preoperative chemotherapy was defined as chemotherapy delivered within the 3 months before metastasectomy and not given in the adjuvant setting after resection of their primary colonic lesion.

Patients were classified as having received a preoperative regimen based on oxaliplatin, irinotecan, capecitabine, or other. Patients were allowed to receive their preoperative chemotherapy outside the institution for added convenience to the patient. All patients were required to receive clinical evaluation by the Department of Thoracic Surgery and all diagnostic imaging at MD Anderson Cancer Center. The resected pulmonary tumor size was defined as the maximal diameter on a computed tomography scan in centimeters. In the case of multiple pulmonary metastases, the diameter of the largest lesion was noted to represent the tumor size. The institutional review board granted an approval for this study with a waiver of patient consent.

### Survival Rate and Statistical Analysis

We performed univariate and multivariate analyses to determine the effect of the different classes of chemotherapy regimen and various clinical variables on progression-free survival (PFS) and overall survival (OS). PFS is defined as time from date of intervention for pulmonary metastases (which will be the date of pulmonary metastasectomy surgery in the surgery alone arm, and the date of start of preoperative chemotherapy in the preoperative chemotherapy arm) to the date of first documented recurrence at any location and OS is defined as the time from the date of intervention for pulmonary metastases (as defined above) to the date of death; patients lost to follow-up or alive at the time of this analysis were censored for the date of last contact.

The multivariate analysis using the Cox proportional hazards model after backward stepwise Wald elimination was conducted using the chemotherapy regimen and clinical variables found to be significant on univariate analysis, defined as a probability value of less than 0.25. A *p* value of less than 0.05 on multivariate analysis was considered significant. We used Kaplan-Meier curves, Cox regression analysis and Mantel-Cox pairwise comparisons to compare the PFS and OS among the groups of patients receiving various chemotherapy regimens using the subset of patient receiving an irinotecan-based regimen or the surgery alone arm as the reference group as specified. All analyses were conducted using SPSS version 19.0 (SPSS Inc, Chicago, IL).
